# Role of Myostatin in Rheumatoid Arthritis: A Review of the Clinical Impact

**DOI:** 10.3390/diagnostics14111085

**Published:** 2024-05-23

**Authors:** Fabiola Gonzalez-Ponce, Melissa Ramirez-Villafaña, Eli Efrain Gomez-Ramirez, Ana Miriam Saldaña-Cruz, Sergio Gabriel Gallardo-Moya, Norma Alejandra Rodriguez-Jimenez, Heriberto Jacobo-Cuevas, Cesar Arturo Nava-Valdivia, Felipe Alexis Avalos-Salgado, Sylvia Totsuka-Sutto, Ernesto German Cardona-Muñoz, Edgar Ricardo Valdivia-Tangarife

**Affiliations:** 1 Instituto de Terapeutica Experimental y Clínica, Programa de Doctorado en Farmacología, Departamento de Fisiología, Centro Universitario de Ciencias de la Salud, Universidad de Guadalajara, Guadalajara 44340, Mexico; fabiola.gonzalez@academicos.udg.mx (F.G.-P.); melissa.ramirez@academicos.udg.mx (M.R.-V.); dr.efrain.gomez@gmail.com (E.E.G.-R.); ana.saldanac@academicos.udg.mx (A.M.S.-C.); norma.rodriguezj@academicos.udg.mx (N.A.R.-J.); sylvia.totsuka@academicos.udg.mx (S.T.-S.); cameg1@gmail.com (E.G.C.-M.); 2 Programa de Doctorado en Farmacología, Centro Universitario de Ciencias de la Salud (CUCS), Universidad de Guadalajara, Guadalajara 44340, Mexico; sergio.gallardo@alumnos.udg.mx (S.G.G.-M.); felipe.asalgado@alumnos.udg.mx (F.A.A.-S.); 3 Programa de Postdoctorado, Departamento de Psicología Básica, Centro Universitario de Ciencias de la Salud, Universidad de Guadalajara, Guadalajara 44340, Mexico; hjacobocuevas@gmail.com; 4 Departamento de Microbiología y Patología, Centro Universitario de Ciencias de la Salud, Universidad de Guadalajara, Guadalajara 44340, Mexico; cesar.navavaldi@academicos.udg.mx; 5 Departamento de Neurociencias, Universidad de Guadalajara, Guadalajara 44340, Mexico

**Keywords:** myostatin, myokines, rheumatoid arthritis, radiographic progression, inflammation

## Abstract

Rheumatoid arthritis (RA) is a chronic inflammatory disease that affects synovial joints and that frequently involves extra-articular organs. A multiplicity of interleukins (IL) participates in the pathogenesis of RA, including IL-6, IL-1β, transforming growth factor-beta (TGF-β), and tumor necrosis factor (TNF)-α; immune cells such as monocytes, T and B lymphocytes, and macrophages; and auto-antibodies, mainly rheumatoid factor and anti-citrullinated protein antibodies (ACPAs). Skeletal muscle is also involved in RA, with many patients developing muscle wasting and sarcopenia. Several mechanisms are involved in the myopenia observed in RA, and one of them includes the effects of some interleukins and myokines on myocytes. Myostatin is a myokine member of the TGF-β superfamily; the overproduction of myostatin acts as a negative regulator of growth and differentiates the muscle fibers, limiting their number and size. Recent studies have identified abnormalities in the serum myostatin levels of RA patients, and these have been found to be associated with muscle wasting and other manifestations of severe RA. This review analyzes recent information regarding the relationship between myostatin levels and clinical manifestations of RA and the relevance of myostatin as a therapeutic target for future research.

## 1. Introduction

Skeletal muscle has recently been recognized as an endocrine organ that is capable of secreting several molecules, including cytokines and myokines (proteins synthesized and secreted by myocytes in response to muscle contraction) [1]. Myokines have an autocrine function involved in regulating muscle metabolism, but they also demonstrate paracrine/endocrine action and can affect other tissues such as bone, adipose, liver, and brain [1]. Myokines are affected both by physical exercise and by inflammatory processes, such as those observed in autoimmune diseases [2,3].

Rheumatoid arthritis (RA) is a chronic inflammatory disease that affects synovial joints and that frequently involves extra-articular organs, affecting 1% of the population worldwide [4]. In RA, the inflammatory processes involve mainly the synovial membrane of the joints, although, frequently, extra-articular tissues are also affected [5]. RA predominantly affects women, with a prevalence ratio of 2–3:1 [4]. In many patients, RA progresses, leading to several grades of permanent functional disability and structural sequelae [6]. Moreover, RA is associated with a decreased life expectancy [7].

RA is a multicausal disease wherein genetic factors are responsible for approximately 50% of the risk of developing the disease, and environmental factors are also involved [8,9]. Among the environmental factors, smoking has been reported as a cause, and mainly affects patients with RA who have seropositivity to rheumatoid factor or ACPAs [10]; exposure to silica [11] and dietary factors, such as the excessive consumption of red meat and coffee and the low consumption of fruits and blue fish, have also been described as risk factors [12,13,14]. The presence of certain infections can also trigger the pathogenesis of RA, such as Lyme disease, Porphyromonas gingivalis, and the Epstein–Barr virus [15,16,17]. In recent studies, a history of COVID-19, as well as a history of having the COVID-19 vaccine administered, has been linked to the development of some types of undifferentiated arthritis, so it is currently being debated whether these conditions are also risk factors for developing RA [18,19].

To date, the pathophysiological mechanisms of RA are not completely clear; however, it is known that the processes begin before the onset of RA symptoms [20]. Among these processes, genetic and environmental interactions occur where modified autoantigens such as immunoglobulin G (IgG), vimentin, and type II collagen are formed. As these antigens contain arginine residues, they can undergo citrullination via the enzyme peptidyl arginine deiminase, then being converted to citrulline [21,22].

In the presence of the human leukocyte antigen-DR isotype (HLA-DR)1 and HLA-DR4, genes present in patients with RA, the immune system’s recognition of citrullinated proteins (vimentin, fibrin, and type II collagen, among others) as the body’s own proteins is blocked [23]. In the presence of these proteins, dendritic cells initiate the immune response, activating T cells, CD4 helper T cells, and B cells. These B cells initiate the production of autoantibodies, which attack the entire body without discriminating against the cells themselves [24]. Of these antibodies, the most studied are rheumatoid factor, which is an IgM with a specificity of 85% in detecting RA [25], and ACPAs, which are more specific for RA, since they target citrullinated proteins, binding to them in order to subsequently maintain this complex in the synovial fluid [26]. When these antibodies are found in the synovial fluid, they cause the expansion of cells, like fibroblasts and macrophages, invading the periarticular bone in the cartilage–bone junction, called “pannus”; in the long term, this causes bone erosion and cartilage degradation [5].

On the other hand, synovial macrophages release various cytokines that participate in the symptoms of RA pathology, mainly interleukin (IL)-1, IL-1β, IL-6, and tumor necrosis factor (TNF)-α, which enhance inflammation through the nuclear factor kappa (NF-k) β pathway [27,28], although IL-12, IL-15, IL-18, IL-17, IL-23, and transforming growth factor-beta (TGF-β) also participate in the physiopathogenesis [29]. Together, all of these molecules and mechanisms cause a pro-inflammatory environment in patients with RA, reflected not only as persistent inflammation in peripheral joints, such as in the hands, feet, and wrists, but also in ways that cause the patient to present with systemic inflammation that can affect various tissues and organs [30]. These conditions, better known as extra-articular manifestations, can occur at any age and are associated with a high risk of premature death because of cardiovascular and pulmonary diseases [31]. The frequency with which extra-articular manifestations occur varies, but they occur in around 40% of patients with RA [32]. Generally, these manifestations are related to more active and severe RA, which is why these patients also usually present high titers of rheumatoid factor (RF) [30]. Among the manifestations are rheumatoid nodules, episcleritis, pleural and pericardial effusions, fibrinoid necrosis of the vessel wall, acute myocardial infarction, myocarditis with rheumatoid nodules, myocardial fibrosis, ulcerative lesions of the lower extremities, keratoconjunctivitis sicca, xerostomia, mesangial glomerulonephritis due to iatrogenic causes, peripheral neuropathy, and hematological abnormalities, with anemia being the most common manifestation [30].

Among the multiple extra-articular manifestations in RA, muscle wasting and rheumatoid cachexia are some of the severe problems that affect patients [33]. Muscle wasting is characterized by a progressive loss of muscle mass and changes in the composition of the skeletal muscle fibers, resulting in decreased strength and function disability [34]. In rheumatoid cachexia, this process of muscle wasting can be accompanied with an increase in or conservation of fat mass and, mainly, with an increase in muscle inflammatory biomarkers [35]. Several myokines released by the muscle during muscle contraction, which have a direct relationship with muscle size, have been identified [36]. Although multiple myokines have been identified, one of the best known in terms of its functioning and clear evidence is myostatin, which is a negative regulator of muscle growth [36].

Currently, experts of musculoskeletal diseases have suggested the importance of conducting clinical trials to evaluate the development of pharmacological interventions that allow the management of sarcopenia through the identification of related biomarkers [37]. Sarcopenia is a condition that has been linked to negative outcomes, including reduced mobility, a low quality of life, hospitalization, and death, in addition to high costs [38,39]. Furthermore, patients with RA have a prevalence of sarcopenia that ranges between 17.1% and 60% [40]. Therefore, it is important to identify biomarkers that allow the evaluation of the effects of pharmacological treatment aimed at managing sarcopenia in RA, in addition to imaging techniques; we define a biomarker as “a characteristic that is objectively measured and evaluated as an indicator of normal biological processes, pathogenic or pharmacological responses to a therapeutic intervention” [37,41]. Myokines are useful for interpreting the pathophysiological mechanisms related to musculoskeletal diseases, as well as the way in which drugs aimed at treating them act, since they are involved with metabolism, angiogenesis, and inflammation, and all of these have impacts on the autocrine, paracrine, and endocrine systems [37,42]. In this sense, myostatin is possibly the most studied myokine, since, in other diseases, it has been found to be a good predictor of mortality. An example of this can be observed in hemodialysis, wherein myostatin allows for the clinical prediction of 1-year mortality [43]. However, regarding the role of myostatin in muscle atrophy, there are many contradictory data that may be due to the analytical heterogeneity of studies regarding age, sex, and the type of musculoskeletal disease in question [37].

However, recent experimental studies have found an association between myostatin levels and molecules involved in the pathogenesis of RA, which is why there has been recent interest in investigating the behavior of this molecule in patients with RA [44,45]. To date, various mechanisms have been described by which myostatin can negatively regulate muscle growth, which can be, in turn, blocked by physical exercise, as well as mechanisms by which myostatin can affect bone integrity [46]. Additionally, it has been described that myostatin values are higher in patients with RA, both clinically and in experimental models [44,45,47]. However, few studies have been performed evaluating the behavior of myostatin in patients with RA, with the aim of describing whether the behavior of myostatin at an experimental level, and that documented in patients with other diseases, is the same as in patients with RA.

Due to the potential role that myostatin has as a biomarker in future studies, it is important to conduct more studies that allow us to understand the behavior of this molecule in patients with RA, allowing us to able to create drugs that act on the myostatin signaling pathway, with the precise dose to be able to counteract the harmful effects, without eliminating the muscle turnover effects and other side effects that may occur. To have a more complete view of the current state of knowledge of serum myostatin levels in rheumatoid arthritis patients, and to identify specific gaps in the knowledge concerning this area, we conducted this review of the current literature. For this reason, the objective of this review is to describe studies on serum myostatin levels in patients with RA that exist to date, with the aim of providing basic knowledge on their clinical relevance for future research.

## 2. Myokines in Rheumatoid Arthritis: Relevance of Myostatin

Myokines are proteins which are secreted mainly by myocytes of skeletal muscle tissue that have diverse functions, including the modulation of various metabolic pathways related to carbohydrates, as well as lipids in adipose tissue and various pro-inflammatory processes [29] (Table 1). Myokines modulate the proliferation, differentiation, and regeneration of muscle fibers in the presence or absence of physical exercise [47]. In addition, myokines collaborate with the communication that exists between the muscle and other organs, such as adipose tissue; the intestine, brain, pancreas, and liver; endothelial tissue; bones; and skin [47]. In the muscle tissue, there are also infiltrating immune cells and muscle macrophages that contribute to the secretion of both cytokines and myokines [48]. Myostatin is one of the best-known myokines because it was the first substance secreted by skeletal muscle tissue to be identified and defined as a myokine [49]. Myostatin is also abundantly expressed in skeletal muscle and has been shown to have multiple endocrine effects [50].

Myostatin is a myokine member of the TGF-β superfamily and is considered a growth differentiation factor, inhibiting the proliferation and differentiation of skeletal muscle cells, and which is also responsible for negatively regulating the growth and development of muscle fibers and limiting their number and size [29]. The effects of myostatin are mediated by the activin receptor type IIB (ActRIIB) [51]. Upon myostatin binding to its receptor, mothers against decapentaplegic (Smad)2 and Smad3 are phosphorylated to form a complex with Smad4, which, in turn, stimulates members of the class O of *forkhead box transcription factors* (FoxO)-dependent transcription [52]. FoxO regulates genes related to the proliferation, differentiation, and degradation of mature myofibrils through the ubiquitin proteasome system [52]. On the other hand, myostatin suppresses the mammalian target of the rapamycin (mTOR) signaling pathway mediated by serine/threonine protein kinase (Akt), thus inhibiting the synthesis of new proteins in muscle tissue [53].

**Table 1:** Myokines released by skeletal muscle and their role in rheumatoid arthritis.

Myokine	Role	Reference
Myostatin/GDF-8	Muscle growth suppressor. Cachexia, bone inflammation mediated by TNF-α, activation of osteoclast formation mediated by RANKL in RA.	Akash, 2023 [54]
Follistatin	Antagonist of TGF-β ligands. Positively correlates with muscle mass. Has improved synovitis and inhibited the erosion of proteoglycans in an experimental study.	Yamada, 2014 [55]
Activin A	Member of the TGF-β superfamily. Negative regulator of muscle growth. Same pathway as myostatin, but cohort-based evidence is still lacking. Contributes to synovial inflammation and pain.	Dong, 2014 [56]
GDF-15	Member of the TGF-β superfamily. It is associated with joint involvement and atherosclerosis in RA.	Tanrikulu, 2017 [57]
Irisin	Responsible for the darkening of white adipose tissue. Low titers are associated with high disease activity, disability, and subclinical atherosclerosis in RA.	Samar, 2002 [58]

Abbreviations: GDF: growth and differentiation factor; TNF-α: tumor necrosis factor-α; RANKL: receptor-associated nuclear factor-κB ligand; TGF-β transforming growth factor-+β; RA: rheumatoid arthritis.

## 3. Myostatin in Rheumatoid Arthritis

As previously noted in this review, various interleukins participate in the pathogenesis of RA, such as TNF-α, IL-1β, and IL-6, among others [28], as RA is a disease in which many of its comorbidities are associated with metabolic problems or muscle wasting, which contribute to functional disability and a worse disease prognosis [33,34,35]. Therefore, several authors have been interested in studying molecules that simultaneously affect all these factors. One of these molecules that participates in all these aspects, but that has been little studied at a clinical level, is myostatin [44,45].

To date, the role that myostatin has in inflammation has been described; it has been found that myostatin positively regulates the expression of TNF-α through the PI3K-Akt and IL-1β pathway in fibroblasts, in addition to inducing the migration of Th-17 cells at the joint level in RA [44,45]. However, the inflammatory role of myostatin, as well as its negative regulation of muscle growth, are not the only aspects that have been described in RA. In the following sections, we review the clinical studies that have been published to date and their main findings in relation to the impact that myostatin levels have on RA disease, as well as what the authors propose, based on their results, to improve the quality of care for patients with RA.

### 3.1. Serum Myostatin Levels in RA Patients Compared to Healthy Controls

In the study performed by Kerschan-Schindl et al., which included 24 women with RA in remission and compared their serum myostatin levels with 24 healthy control women [59], serum myostatin levels, obtained using colorimetric competitive immunoassay, were higher in age-matched healthy control patients than in RA patients in remission (according to the Clinical Disease Activity Index (CDAI), with a median of 1.0 (0.4–1.6)) (myostatin levels: 37.4 (29.9–44) ng/mL vs. 49.7 (45.3–57.3) ng/mL; *p*-value < 0.001) [59]. Additionally, it was found that serum myostatin levels correlated inversely with physical function (evaluated using the Health Assessment Questionnaire, specific for diseases that affect daily activities) [60]. The following negative correlation values were found between myostatin and left hand grip strength (r = −0.370, *p* < 0.001) and myostatin and right hand grip strength (r = −0.318, *p* < 0.05), with a positive correlation found between myostatin and the Health Assessment Questionnaire (r = 0.494, *p* < 0.001); the higher myostatin levels were, the worse the physical functionality of patients with RA [59].

Murillo-Saich et al. compared the serum myostatin levels (measured using an enzyme-linked immunosorbent assay) of 84 women with RA experiencing different degrees of disease activity (a disease activity score in 28 joints (DAS28) of 2.8 (0.9–7.1)) and 127 women without rheumatic disease, finding higher levels of myostatin in the RA group (9 (1.2–140) ng/mL) than in the group without rheumatic disease (3.5 (1–89.9) ng/mL) [61]. Additionally, serum myostatin levels presented a direct correlation with the duration of RA (13 (0–45) years of duration) (r = 0.24, *p* = 0.02), with C-reactive protein (CRP) (r = 0.40, *p* < 0.001), with the erythrocyte sedimentation rate (ESR) (r = 0.28, *p* = 0.009), as well as with the total dose of glucocorticoids (r = 0.51, *p* < 0.001) [61]. In this study, the control group was made up of women of similar ages and body mass indexes (BMIs) to those of the RA group, but without the presence of inflammatory or autoimmune diseases, chronic kidney diseases, cancer, and active infections; however, not all of them were healthy. High blood pressure occurred in 33% of the patients, and type II diabetes mellitus occurred in 22%; nevertheless, these comorbidities occurred in a similar way to the RA group [61].

Similarly, a study conducted by Lin et al. in China compared the myostatin levels of 344 patients with RA vs. 118 healthy controls and reported higher myostatin levels in the RA group at the baseline of the study (3.241 ± 1.679 ng/mL, vs. 1.717 ± 0.872 ng/mL, respectively; *p* < 0.001) [62].

Meanwhile, a study performed by Gonzalez-Ponce et al. in Mexico evaluated 161 women with RA and compared their serum myostatin levels (obtained using enzyme-linked immunoassay) with 72 women without rheumatic disease (but including those with high blood pressure and diabetes mellitus type II). This study, similarly, found higher serum myostatin levels in the RA group (11.89 (1.2–140) ng/mL) than in the control group (7.9 (1.2–19.6) ng/mL) [63] (Table 2).

### 3.2. Effects of Exercise on Serum Myostatin Levels in Patients with RA

A study performed by Andonian et al. in the United States of America evaluated the effects of high-intensity interval training for 10 weeks on serum myostatin levels in 12 patients with RA (both genders), comparing them with the changes achieved using the same type of exercise over the same duration in nine patients with pre-diabetes mellitus [64]. Patients with pre-diabetes included in this study had to meet the following parameters: hemoglobin A1C between 5.7% to 6.5%, stable use of all medications for at least 3 months, and initial exercise less than 2 times per week. In addition to this, patients who already had an established diagnosis of diabetes and the inability to walk without assistance were excluded [64]. The clinical characteristics of the patients with RA at the beginning of the study were as follows: they had an RA duration of 13.3 ± 7.2 years; positive rheumatoid factor (RF) was present in 83.3% of the patients; 62.5% were positive for anti-cyclic citrullinated antibodies (anti-CCP); and radiographic erosions were present in 75% of the participants [64]. Any joint complications or extra-articular complications they presented with were not described. At the beginning of the study, the patients with RA presented a disease activity score in 28 joints (DAS-28) of 3.1 ± 1.3, while, after the high-intensity interval training program, the score decreased to 2.3 ± 1.5, with a statistically significant change (*p* < 0.05) [64]. Patients underwent biopsies of the vastus lateralis, the skeletal muscle of which was homogenized, and the sample concentrations were quantified using an enzyme-linked immunosorbent assay (ELISA) to obtain the myostatin levels. RA patients had a baseline myostatin level of 16.6 ± 7.5 ng/mL [64].

After 10 weeks of high-intensity interval training, the levels increased to 20.6 ± 8.7 ng/mL; however, these changes were not significant (*p* > 0.05). In patients with pre-diabetes mellitus, the levels obtained both pre-training and post-training were 31.9 ± 14.3 ng/mL vs. 34.3 ± 20.1 ng/mL (*p* > 0.05), respectively, and these changes were statistically significant [64]. In this study, it was observed that, after 10 weeks of high-intensity interval training, intramuscular cytokines were modified, while the amount of lean mass increased and the percentage of body fat decreased; however, this improvement was less evident in patients with RA when compared to patients with prediabetes mellitus when performing the same training [64]. These results make it clear that abnormal muscle remodeling occurs in RA despite exercise, so it is important to better understand the interaction between disease-modifying therapy, cardiorespiratory fitness, exercise, and body composition in order to improve the health of people with disabilities and reduce the risk of cardiovascular disease (CVD) [64].

### 3.3. Radiographic Progression Is Associated with Serum Myostatin Levels in RA Patients

In the study performed by Lin et al., they evaluated serum myostatin levels (measured using an enzyme-linked immunosorbent assay) at the beginning of the study, and, according to the average level obtained, divided patients into low serum myostatin levels (174 patients) (<2841 ng/mL) and high myostatin levels (172 patients) (≥2841 ng/mL) [62]. These values were obtained according to the median level of serum myostatin at the baseline [62]. The group with higher myostatin levels had a higher rate of active smoking (20% vs. 9.9%), a higher body mass index (22.2 ± 3.4 kg/m 2 vs. 21.5 ± 3.2 kg/m^2^), and a higher radiological evaluation rate, as assessed using the modified total Sharp score (mTSS), (median mTSS 14 vs. 6), but a lower proportion of women (77.9% vs. 90.1%) and a lower history of biological agents (7% vs. 16.3%) [62]. The mTSS is a system used to quantify radiological changes in patients with RA through the erosion and narrowing of 27 small joints [65]. The erosion is scored from zero to five points (one = discrete changes, two to three = greater changes; a score of >three indicates the size of the erosions). The narrowing of the joint space is scored from zero to four points (zero = normal space, one = suspect narrowing, two = global narrowing of <50% of the original space, three = global narrowing of >50% of the original space or subluxation, and four = articular ankylosis or total luxation) [65]. After 12 months of follow-up, RA patients with high serum myostatin levels presented a higher rate of radiographic progression (45.3% vs. 18.6%), as assessed by the modified total Sharp score (mTSS), joint space narrowing (JSN), and joint erosion (JE) [62].

Additionally, these groups were each subdivided into non-myopenia and myopenia, reporting that the high myostatin–myopenia group had the lowest frequency of women (77.6% vs. 90.6%, *p*-value 0.023), the highest frequency of active smokers (23.9% vs. 8.2%, *p*-value 0.033), the shortest duration of RA (66 (27–120) vs. 71 (23–96) months, *p*-value 0.007), the highest pain score according to the visual analogue scale (VAS) (4 (4–5) vs. 2 (2–4), *p*-value 0.028), the highest scores on the Health Assessment Questionnaire Disability Index (HAQ-DI) (0.3 (0.0–1.0) vs. 0.3 (0.0–0.8), *p*-value 0.019), and a lower frequency of use of biological agents (7 (10.4%) vs. 17 (20%), *p*-value 0.013) [62]. Using logistic regression, the risk in patients to have one-year radiographic progression was calculated. The result was that patients were at greater risk when high levels of myostatin were present together with myopenia (OR = 1.432 (0.528–3.887), *p* = 0.481; high myostatin overlapping non-myopenia: OR = 3.084 (1.314–7.241), *p* = 0.010; high myostatin overlapping myopenia: 10.425 (3.959–27.450), *p* < 0.001) [62]. In this study, myopenia was diagnosed via bioelectrical impedance analysis, being defined as an appendicular skeletal muscle mass index (ASMI) of ≤7.0 kg/m^2^ in men and of ≤5.7 kg/m^2^ in women, according to the Asian Task Force for Sarcopenia [66]. The HAQ-DI score was obtained using a questionnaire, which evaluated the functional disability in RA patients through nine items related to the difficulty of performing daily activities [67].

### 3.4. Appendicular Skeletal Muscle Mass Index Associated with Serum Myostatin Levels in RA Patients

In the study performed by Murillo-Saich et al. [61], they found an inverse correlation between myostatin serum levels, skeletal muscle mass index, and free fat mass index, while, in the study by Gonzalez-Ponce et al. [63], the group of RA patients with low muscle mass also had higher levels of myostatin, as well as a greater frequency of patients with high levels of myostatin. Both studies agree with the hypothesis since myostatin is a negative regulator of muscle growth [61,63]. To determine the presence of low muscle mass or myopenia, the segmental lean mass was first obtained through the muscle mass of the arms and legs, evaluated using dual X-ray absorptiometry (DXA), and the skeletal muscle mass index (SMMI) was calculated using the formula ((arms + legs)/adjusted for height in square meters) [68]. Myopenia was considered present if the SMMI score was <5.5 kg/m^2^ [69]. Cachexia was considered present if the patient presented a decreased fat-free mass index (FFMI), but also a stable or increased fat mass [35]. The FFMI appeared low when it was below the 10th percentile, and the fat mass index (FMI) appeared low when above the 25th percentile of the reference population [70]. In this population, the 10th percentile of the FFMI was 13.74 kg/m^2^, and the 25th percentile of the FMI was 11.27 kg/m^2^ [63].

Nevertheless, in the study by Lin et al., a Spearman correlation was also performed between the serum myostatin levels and muscle mass, evaluated using the appendicular skeletal muscle mass index (ASMI), with a positive correlation being found between these two variables (r = 0.230, *p* < 0.001) [62]. At the baseline, 44.2% of RA patients had myopenia, while 21.2% of healthy subjects in the control group had myopenia [62]. RA patients with myopenia showed a lower level of serum myostatin at the baseline when compared with those who did not have myopenia (3004 ± 1640 ng/mL vs. 3428 ± 1689 ng/mL, *p* = 0.013) [62] (Table 3).

### 3.5. Myostatin Cutoff

In the study performed by Gonzalez-Ponce et al., they determined a myostatin value of ≥17 ng/mL as an independent factor for muscle mass risk (OR = 3.04, 95% CI 1.14–8.10), with a sensitivity of 43%, indicating the possibility of positive myostatin results of ≥17 ng/mL in patients with myopenia, and a specificity of 77%, indicating the probability of negative myostatin results ≥17 ng/mL in RA patients without myopenia. The same cut-off point presented a sensitivity of 53% and a specificity of 71% as a risk factor for presenting rheumatoid cachexia (OR = 2.79, 95% CI 1.17–7.89) [63].

In this study, Engvall’s criteria were used to define rheumatoid cachexia, which is the loss of skeletal muscle mass, determined by a fat-free mass index (FFMI) value below the 10th percentile when compared to the reference population, but with stable or increased fat mass, as determined by a fat mass index (FMI) value above the 25th percentile, according to the reference population [70]. In the study by Gonzalez-Ponce et al., the reference population had an FFMI 10th percentile of 13.74 kg/m2, and an FMI 25th percentile of 11.27 kg/m^2^ [63].

The study performed by Murillo-Saich et al. found a myostatin cut-off point of ≥13 ng/mL to be a risk factor for presenting moderate/severe disease activity (OR = 2.93, 95% CI 1.13–7.61), with a sensitivity of 61%, indicating the possibility of positive myostatin results in patients with moderate/severe disease, and a specificity of 53%, indicating the possibility of negative myostatin results in RA patients with low activity/remission [61]. The disease activity was evaluated using DAS28, which is an index that is obtained via evaluating the following four parameters: the number of painful joints among 28 joints, the number of swollen joints among 28 joints, the subjective global evaluation reported by the patient on a scale from 0 to 100, and the C-reactive protein (CRP) value or the erythrocyte sedimentation rate (ESR) [71]. After the evaluation, the DAS28 index was calculated, and the results allowed the disease activity of the patient with RA to be classified as in remission <2.6, mild 2.6–<3.2, moderate > 3.2–5.1, or severe activity > 5.1 [72] (Table 4).

## 4. Discussion

In the present review, it is evident that discrepancies persist in the findings of the studies previously mentioned. In the studies performed by Lin et al., Murillo-Saich et al., and Gonzalez-Ponce et al., the patients with RA had higher levels of myostatin when compared to control groups without rheumatic disease [61,62,63]. Although in the studies of Murillo-Saich et al. and Gonzalez-Ponce et al., only women with RA were evaluated, in the study of Lin et al., both sexes were evaluated. The samples evaluated in these three studies had several features in common; specifically, patients with RA had a low prevalence of the use of biological agents, and patients who had active RA were included, according to the DAS28 score [61,62,63]. On the other hand, in the studies carried out by Murillo-Saich et al. and Gonzalez-Ponce et al., more than half of the patients were sedentary, possibly a secondary effect of the symptoms of active RA [61,63]. A sedentary lifestyle was defined as individuals who did not perform at least 150 min of moderate aerobic physical exercise per week [73].

In the findings reported by Kerschan-Schindl et al., the presence of lower serum myostatin levels in patients with RA when compared to healthy controls could be associated with the fact that all patients with RA were in remission [59]. Another important characteristic was that almost all patients were treated with biological agents, whose immunomodulatory effects could have positive effects on the reduction in myostatin; however, high serum levels of myostatin correlated with a low grip strength in both hands, as well as worse functional disability [61]. In this study, it was not reported what percentage of patients with RA were sedentary, so this is a parameter that cannot be considered as a factor [61].

It had previously been reported that RA is characterized by an excess of proinflammatory cytokines, as well as a deficit in muscle mass, so these causes were investigated in an in vitro study, which found that myostatin regulated IL-1β expression through the transduction pathways of extracellular signal-regulated kinase (ERK), mitogen-activated protein kinases (JNK), and activating protein-1 (AP-1) signals [74]. The IL-1β produces bone resorption in RA and increases joint inflammation, which is why it is related to greater disease activity [75,76]. For this reason, it could be believed that myostatin aims to be a potential therapeutic target for RA [74].

**Figure 1:**
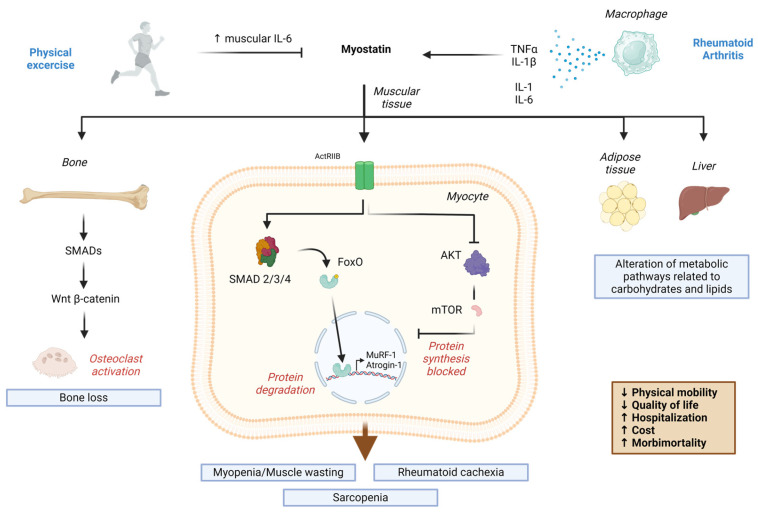
Proposed mechanism considering the experimental contributions and effects of myostatin at the clinical level. The figure describes the release of myostatin from muscle myocytes, which is blocked by physical exercise, while physical exercise temporarily promotes the release of IL-6 by skeletal muscles. According to studies described in this review, levels of IL-6 secreted by muscle are inversely associated with myostatin levels. In RA, myocytes release interleukins, of which TNF-α and IL-1β stimulate the greater production of myostatin. Myostatin-mediated bone destruction is carried out by SMADs activating the Wnt/β-Catenin pathway that promotes the production of osteoclasts in the synovial membrane. Muscle wasting occurs through the ActRIIB receptor, which activates the UPS through FoxO, inducing muscle degradation, and, on the other hand, inhibits the AKT/mTOR pathway, preventing the formation of new muscle, a condition that results in myopenia. In adipose tissue and liver, myostatin alters the metabolism of carbohydrates and lipids, which triggers an increase in adipose tissue. The increase in adipose tissue added to low muscle mass results in rheumatoid cachexia. Abbreviations: SMAD: mothers against decapentaplegic, ActRIIB: activin receptor type IIB, FoxO: Members of the class O of *fork head box transcription factors*, UPS: ubiquitin–proteasome system, Akt: serine/threonine protein kinase, mTOR: mammalian target of rapamycin, IL: interleukin. This figure was made with Bio Render (Agreement number: JS26RMD8OB).

Regarding the effects that high-intensity physical exercise has on myostatin levels in patients with RA, Andonian et al. reported that muscle myostatin did not demonstrate significant changes after 10 weeks of training, although disease activity, as assessed using DAS-28, did show significant improvement [64]. On the other hand, muscle IL-6 levels did decrease, which may explain the reduction in disease activity in RA, and this reduction was associated with an increased lean mass and lower fat mass [64]. Under normal conditions, it has been described that, during and after exercise, the serum levels of IL-6 increase temporarily, in the same way that it has been reported that myostatin is negatively regulated by exercise [77,78]. However, in the study performed by Andonian et al., myostatin levels increased after exercise in the group of patients with RA, which shows that the myostatin pathway was altered [64]. This can be explained by the decrease in IL-6 levels that Andonian et al. report to occur after exercise, since, in other conditions, IL-6 levels are inversely correlated with myostatin, which is why, in this case, it may have been the cause of the increase; however, the precise path should be studied further [79] (Figure 1).

These results can be explained by the relationship between myostatin and IL-6, which has already been described experimentally; myostatin, through the p38MAPK pathway, increases the levels of IL-6, while IL-6 leads to the stimulation of the suppressor of cytokine signaling (SOCS3), which degrades the insulin receptor substrate (IRS)-1, inhibiting insulin/insulin growth factor (IGF)-1, Akt, and p-FoxO signaling, which are essential pathways for proteogenesis, meaning that protein synthesis is reduced [80]. However, these effects were more noticeable in the comparison group (prediabetes) than in RA, even though both diseases have a similar risk of cardiovascular diseases [64]. The reduction in IL-6 after training occurred at the muscle level, but, since RA is a systemic inflammatory disease, the production of IL-6 continued to occur at the joint level [64,81]. It has been described, in the pathogenesis of RA, that the excessive production of IL-6 activates the JAK pathway, which in turn activates STAT3 [82]. This pathway stimulates proteolysis through the ubiquitin proteasome system by activating MuRF-1 and Atrogin-1/MAFbx at the nuclear level [82]. The impact of IL-6 on muscle degradation was investigated in a study in which tocilizumab, an anti-IL-6 receptor antibody, was used for 1 year in patients with RA; these patients saw an increase in muscle mass as compared to the muscle mass that they had at the beginning of the study [83].

Regarding pharmacological treatment, it was found that those who used a monoclonal antibody against the IL-6 receptor presented an increase in muscle mass without changes in their fat percentage, unlike those who took anti-TNF [64]. For this reason, in the study performed by Andonian et al., they suggested, based on their findings, that IL-6, which is persistently elevated in RA, alters the normal mechanisms of muscle adaptation to physical exercise [64]. It had previously been described that systemic inflammation is persistent in patients with RA, since, under normal conditions, serum IL-6 is reduced after chronic training; however, in these patients, resistance to IL-6 occurs secondary to chronic inflammation [84]. However, if this chronic inflammation is accompanied with physical inactivity, physical inactivity can cause an increase in visceral fat, which in turn contributes to the secretion of a greater number of cytokines, producing a higher level of chronic inflammation [84].

There is evidence that exercise produces changes in the secretion of various interleukins in response to muscle contractions, with IL-6 demonstrating the highest level [78,85]. However, IL-6 levels increase rapidly during exercise, but decrease after the exercise ends [86]. It is important to highlight that the elevation of IL-6 during exercise has favorable effects. Firstly, it has been proven that the increase in IL-6 after exercise derives only from skeletal muscle and not from circulating immune cells [87]; secondly, IL-6 has been shown to inhibit TNF-α production in human monocytes [88]. Thirdly, a low level of muscle glycogen positively stimulates IL-6 expression via regulating hepatic glucose production and increasing lipolysis in adipose tissue [89,90], as well as inducing the expression of the GLUT4 transporter [91]. For all these reasons, the indication of moderate physical exercise to patients with RA is justified, since it can help reduce symptoms of pain and stiffness by reducing systemic IL-6 [78,92]. These findings highlight that, although the implementation of physical exercise is important and reflects an improvement in the quality of life of these patients, it is also important to conduct more studies that better evaluate the side effects of the pharmacological treatment used in these patients with RA on their serum levels and muscle myostatin.

In addition to the autocrine effects of myostatin on the same muscle, the study conducted by Lin et al. found that high serum myostatin is a predictor of joint destruction in patients with RA [62]. The researchers observed that those patients who had high serum myostatin had approximately three times the risk of presenting radiological progression after one year than those with low myostatin levels; however, when these high levels occurred together with myopenia, the destruction of cartilage was even greater than when they occurred in isolation [62].

An experimental study found that myostatin could inhibit the proliferation and differentiation of mesenchymal stem cells [93], while another study demonstrated that it could interfere with osteoblastic differentiation, thus jointly affecting the formation of new cartilage [94]. However, the interaction that occurs between the effects of high myostatin levels with the presence of myopenia is an aspect that requires further research to explain its mechanisms. Myostatin is a negative regulator of muscle mass, but it was not presented in all patients with high myostatin levels who had myopenia, and this combination had a synergistic impact on radiographic progression [62]. Nevertheless, there is already evidence in the experimental phase that myostatin stimulates the formation of osteoclasts in the synovial membrane, which are responsible for bone destruction, through the activation of SMADs and the signaling pathways of protein kinase activated by mitogens, inhibiting the Wnt/B-Catenin pathway, and thus affecting muscle and bone together [95].

In the study conducted by Lin et al., as well as in the study by Gonzalez-Ponce et al., high levels of serum myostatin were related to low scores on the skeletal muscle index in patients with RA [62,63]. Furthermore, in the study by Gonzalez-Ponce et al., serum myostatin levels were also associated with the presence of rheumatoid cachexia [41]. Rheumatoid cachexia is the presence of low muscle mass and is accompanied with an increase in the percentage of body fat [96]. These findings support the negative regulatory role of myostatin on skeletal muscle mass [97]. Low skeletal muscle mass is one of the most important conditions of sarcopenia, which is a condition that does not have a unified definition or diagnosis [98,99]; however, one of the definitions used in RA is that of Baumgartner et al., who considered a diagnosis of sarcopenia to be present when the skeletal muscle index was two standard deviations below the mean of a gender-specific reference group [68]. Subsequently, the European Working Group on Sarcopenia in Older People (EWGSOP) defined sarcopenia as “a syndrome characterized by a progressive and widespread loss of skeletal muscle mass and strength with risk of adverse outcomes such as physical disability, poor quality of life and death” [100]. For this reason, a low muscle mass accompanied with low muscle strength was included in the diagnosis [101].

Sarcopenia occurs in between 10.1% and 45.1% of patients with RA [102,103], and this is a condition of interest because it has the following consequences: an increased risk of falls, fractures, low bone mineral density, cardiometabolic risk, and endothelial dysfunction [83]. Risk factors for sarcopenia in RA include older age, a lower body mass index (BMI), body fat, duration of the disease, bone erosion, joint damage, malnutrition and low protein intake, functional disability, higher protein levels, C reactive (CRP), erythrocyte sedimentation rate (ESR), rheumatoid factor (RF), matrix metalloprotease 3 (MMP3), and glucocorticoid use [83]. However, proinflammatory cytokines, such as IL-6, IL-1β, and TNF-α, could be elevated in the presence of active RA, as well as in functional deterioration. This is related to myopenia as well as rheumatoid cachexia, since these cytokines activate nuclear factor kβ (NF-kβ), and this, in turn, induces proteolysis through the ubiquitination of muscle fibers [104]. However, at an experimental level, it has been found that myostatin promotes the expression of IL-1β in synovial fibroblasts through the ERK, JNK, and AP-1 signal transduction pathways, which is why it is an aspect that requires further investigation in order to elucidate whether myostatin alone is capable of degrading muscle mass without an active disease, or if an active disease degrades muscle mass without the elevation of myostatin [75].

Furthermore, all pro-inflammatory cytokines, and the presence of greater body fat that occurs in rheumatoid cachexia, increase the risk of cardiovascular diseases, promoting an atherogenic environment, which could lead to premature death [97]. However, it is unknown if myostatin could also be involved in this process of increased cardiovascular risk, or if its blockade as a therapeutic target could contribute to improving the quality of the lives of these patients. Nevertheless, it is important to highlight that, when considering myostatin as a therapeutic target, it should not be considered as an isolated biomolecule involved in the process of muscle mass loss. In addition to myokines, the role of vitamin D and its receptors has been reported as playing an important role in the maintenance of skeletal muscle [105]. It has recently been found that patients with RA have lower levels of vitamin D than patients without RA [106]. Additionally, it has been reported that, in patients with RA, low levels of vitamin D are related to severe sarcopenia and poor physical performance [107]. The reason this association exists is because vitamin D participates in the regulation of oxidative stress and calcium homeostasis, the suppression of cytokines and macrophages, and the promotion of the differentiation of regulatory T cells [107]. On the other hand, in a study conducted in mice with induced muscular dystrophy, the inhibition of myostatin through the expression of the follistatin transgene resulted in early improvements in histopathology, but, in the long term, muscle degeneration increased [108]. These results make it clear that, before having myostatin as a possible therapeutic target, many studies will still be required to allow this to be undertaken safely.

It should be noted that the study by Gonzalez-Ponce et al. identified a myostatin cut-off point of ≥17 ng/mL as a risk factor that doubled the risk of developing rheumatoid cachexia and tripled the risk of myopenia for patients with RA [63]. Similarly, the study by Murillo-Saich et al. reported a myostatin cut-off point of ≥13 ng/mL, which doubled the risk of patients presenting with moderate/severe disease activity [61]. These findings could support the hypothesis that myostatin contributes to greater inflammatory processes; however, due to the cross-sectional nature of these studies, it is impossible to attribute causality, so more longitudinal studies are necessary to evaluate whether the control of myostatin levels in patients with RA could also benefit the patient’s cardiovascular health.

These studies, although they contribute knowledge to the area of biomarkers in RA, have several limitations. Because the study carried out by Andonian et al. was a pilot study, it had a low sample size and a short follow-up period; however, when expanded, it could show significant and different changes, as well as heterogeneity in the treatment used [64]. The limitations in the studies by Kerschan-Schindl et al., Murillo-Saich et al., and González-Ponce et al. are related to the fact that they are cross-sectional designs, meaning that they do not allow us to evaluate causality or changes in myostatin levels or the presence of confounding factors related to low muscle mass, as well as having a relatively small number of patients evaluated, which could limit the statistical power [59,61,63]. The limitations in the study performed by Lin et al. included a small proportion of patients without prior treatment; unifying the treatment regimen would serve to eliminate this confounding factor [62]. These limitations are important aspects to consider in future research with the intention of replicating these studies, as taking care to control these factors could improve the contributions in this area.

To date, some pharmaceutical companies have developed myostatin inhibitors, known as small molecules and antibodies, and have classified them into two types as follows: class I, which includes MYO-029, SRK-015, LY2495655, AMG-745/PINTA-745, REGN1033, domagrozumab, and BMS-986089/RO7239361, which are aimed at specifically counteracting myostatin; and class II, which includes ACE-031/ramatercept, bimagrumab, and ACE-083, and is aimed at binding to myostatin receptors and ligands, such as GDF-11 and activin A; these, however, are in clinical phase II or III, so there are still many aspects to be elucidated [109]. However, in the current market, there are already monoclonal and anti-TNF antibodies for the management of RA that, although they are not specific myostatin inhibitors, more studies should be necessary to identify the level of impact on myostatin or muscle mass. Among all monoclonal antibodies, the most used are adalimumab and infliximab, being used as TNF inhibitors, Rituximab as B-cell depleters, and tocilizumab as IL-6 inhibitors [110]. These biopharmaceuticals are used to treat various autoimmune diseases, while, in RA, it has been found that they maintain better therapeutic success when compared to non-steroidal anti-inflammatory drugs, glucocorticoids, or synthetic disease-modifying antirheumatic drugs (DMARDs) [110]; nevertheless, the prevalence of use in patients with RA within the studies analyzed in this review is low. As previously described, myostatin levels are related to IL-6, TNF-α and IL-1β levels in some studies, but the results are inconclusive [44,45,54,81], while the beneficial effect of using the IL-6 inhibitor tocilizumab on muscle mass has already been reported, leading to an increase within 1 year of use [84]. For this reason, it would be interesting to conduct more studies evaluating the effects on muscle, bone, and myostatin levels of various monoclonal antibodies, mostly used in the pharmaceutical market and in health services.

## 5. Conclusions

Myostatin is a myokine that is involved in various muscle processes which, in turn, modulate the production of muscle cytokines. To date, it is a molecule that is still being studied, mainly at a clinical level, since few studies have been performed in humans, and especially in RA patients, because, although it is well known as a negative regulator of muscle growth, it may be involved in other conditions in the pathogenesis of RA, such as inflammation and joint disease, and may even behave differently at the muscular level, depending on the characteristics of the population evaluated.

Therefore, continuing with studies of the behavior of this molecule at a clinical level is very important. It should be noted that the creation of drugs aimed at blocking myostatin should not assume a direct benefit to muscle mass, taking into account that an irreversible action on myostatin increases muscle degeneration in the long term, and the effects of maintaining low long-term levels of myostatin in humans are still unknown. On the other hand, the use of physical activity to improve functional ability in patients with RA continues to be controversial, due to the issue of cytokines. Although the mechanism by which IL-6 is favorable during exercise is known, it would be interesting to know in depth the appropriate exercise time and intensity that enables improvements in the concentration of systemic cytokines, which, in turn, allows myostatin to be blocked at an appropriate intensity for the patient with RA.

## 6. Future Directions

The identification of myostatin as a myokine with roles in both local muscle activity, by negatively regulating muscle growth, as well as in paracrine activity, by affecting the release of cytokines and joint function, could identify myostatin as a therapeutic target in these patients for the purposes of developing myostatin inhibitor pharmacological agents. Currently, some drugs have been developed that are in the evaluation phase in humans, such as stamulumab, with the aim of treating muscular dystrophy, landogrozumab, to treat cachexia, and trevogrumab, to increase muscle mass in healthy subjects [111]; however, the greater identification of the physiological and pathophysiological mechanisms of this molecule may reinforce knowledge in this area and, in turn, generate specific drugs to treat other conditions in patients with RA, such as those described in this review.

## Figures and Tables

**Table 2:** Comparison of serum myostatin levels between patients with rheumatoid arthritis versus subjects without rheumatic disease. Cross-sectional studies.

Author/Country/Year	Groups	Results	Conclusions
Kerschan-Schindl K,/Austria/2019 [59]	- 24 female RA in remission. - 24 female healthy controls	- Female healthy controls have higher myostatin levels (ng/mL) when compared to female RA remission (49.7 (45.3–57.3) vs. 37.4 (29.9–44), *p*-value: <0.001) *** Correlation of myostatin with - Grip strength left: r = −0.370 *p* < 0.01 * - Grip strength right: r = −0.318, *p* < 0.05 * - HAQDI: r = 0.492, *p* < 0.01 * Serum myostatin levels obtained using the colorimetric competitive immunoassay technique.	RA patients in remission have reduced serum levels of myostatin when compared with healthy controls. Patients with higher myostatin levels had a lower grip strength in both hands and worse physical functional condition.
Murillo-Saich JD/Mexico/2021 [61]	- 84 RA - 127 without rheumatic diseases	- Female controls have higher myostatin levels (ng/mL) when compared to female RA (3.5 (1–89.9) vs. 9 (1.2–140), *p*-value: <0.001) *** Serum myostatin levels obtained using the ELISA technique.	Myostatin was associated with disease activity in RA patients, suggesting a mechanistic link between myostatin, muscle wasting, and inflammation in RA.
Lin JZ/China/2022 [62]	- 344 RA - 118 Healthy controls	- RA patients have higher myostatin levels (ng/mL) when compared to controls (3.241 ± 1.679 vs. 1.717 ± 0.872, *p*-value: <0.001) ** Serum myostatin levels obtained using the ELISA technique.	Serum myostatin levels are higher in patients with RA than in healthy controls
Gonzalez-Ponce F/Mexico/2022 [63]	- 161 RA - 72 CL	- RA patients have higher myostatin levels (ng/mL) when compared to controls (11.89 (1.2–140) vs. 7.9 (1.2–19.6), *p*-value: <0.001) *** Serum myostatin levels obtained using the ELISA technique.	Myostatin level are higher in RA female patients.

Table showing the myostatin serum levels observed in patients with RA in cross-sectional studies. Spearman’s correlation (*) was used to identify potential associations with myostatin. The studies compare myostatin levels using the T-Student test (**) when the values are expressed as mean ± SD and use the Mann–Whitney U test (***) when the values are expressed as median and range. Abbreviations: ELISA: enzyme-linked immunosorbent assay.

**Table 3:** Skeletal muscle mass associated with serum myostatin levels in RA patients.

Author/Country/Year	Groups	Results	Conclusions
Murillo-Saich JD/Mexico/2021 [61].	84 female RA	Correlation of myostatin levels with - SMMI: r = −0.29, *p* = 0.008 * - FFMI: r = −0.24, *p* = 0.027 *	Myostatin was inversely related to the index of skeletal muscle mass and fat-free mass.
Lin JZ/China/2022 [62].	344 RA	Myostatin levels (ng/mL) 3004 ± 1640 with myopenia vs. 3428 ± 1689 without myopenia, *p*-value = 0.013 **	RA patients with myopenia had lower levels than the group without myopenia at the beginning of the study.
Gonzalez-Ponce F/Mexico/2022 [63].	161 female RA: - 85 low muscle mass - 75 normal muscle mass	- RA + low muscle mass had higher myostatin levels (ng/mL) when compared to RA + normal muscle mass (13.54 (1.88–140) vs. 10.02 (1.2–117); *p*-Value = 0.02 *** - RA + low muscle mass had higher frequency of high myostatin levels (≥17 ng/mL) when compared to RA + normal muscle mass (37 (43%) vs. 17 (23%); *p*-Value = 0.006) ****	A high myostatin level is a risk factor for low muscle mass.

Table showing the relationship of serum myostatin levels with muscle wasting in patients with RA in cross-sectional studies. Spearman (*) was used to correlate myostatin with the SMMI and FFMI. The studies compare myostatin levels using the T-Student test (**) when the values are expressed as mean ± SD and using the Mann–Whitney U test (***) when the values are expressed as median and range. chi-square tests (****) were used to compare qualitative variables. Abbreviations: SMMI: skeletal muscle mass index; FFMI: fat-free mass index.

**Table 4:** Studies that found a cutoff point for myostatin in RA patients.

Author/Country/Year	Groups	Results	Conclusions
Murillo-Saich JD/Mexico/2021 [61].	84 female RA	Myostatin cut-off (≥13 ng/mL) point associated with moderate or severe disease activity: OR = 2.938 (CI 95% 1.13–7.61, *p*-value 0.027)	Myostatin (≥13 ng/mL) had a sensibility of 61.3% and a specificity of 53% to detect moderate or severe disease activity in RA patients
Gonzalez-Ponce F/Mexico/2022 [62].	161 female RA	Myostatin cut-off point (≥17 ng/mL) associated with low muscle mass: OR = 3.04 (CI 95% 1.17–7.89, *p*-value 0.02).	Myostatin (≥17 ng/mL) had a sensibility of 43% and specificity of 77% to detect low muscle mass in RA patients, and a sensibility of 53% and specificity of 71% to detect rheumatoid cachexia.

Table showing the cut-off points found for myostatin as a risk factor for myopenia, rheumatoid cachexia, and moderate/severe disease activity, with their respective values of sensitivity and specificity in patients with RA. Cutoff value of high levels of myostatin based on ROC curve analysis; odds ratio (OR) estimated using logistic regression.

## Data Availability

Not applicable.
